# Are Ammonia Sensors Ready for Outdoor Use?

**DOI:** 10.1021/acsomega.5c07873

**Published:** 2025-12-29

**Authors:** Pablo Espina-Martin, Sarah R. Leeson, Robert Nicoll, Clare Pearson, Cristina Martin Hernandez, Nathalie Redon, Neil J. Mullinger, Karen Yeung, Marsailidh M. Twigg, Ajinkya G. Deshpande, Matthew R. Jones, Hilary Costello, Graham Spelman, Christine F. Braban

**Affiliations:** † 41865 UK Centre for Ecology and Hydrology, Bush Estate, Penicuik EH26 0QB, U.K.; ‡ Centre for Energy and Environment, IMT Nord Europe, Institut Mines-Télécom, Univ. Lille, F-59000 Lille, France; § Limosaero Limited, 11c Alma Road, Snettisham, King’s Lynn PE31 7NY, England

## Abstract

Ammonia (NH_3_) gas is primarily an agriculture atmospheric pollutant, and measuring near-emission sources is essential for understanding NH_3_ emission plumes. Sensors in theory are attractive alternative monitoring methods due to their high-time data resolution, size, and lower costs; however, there are metrological and technical challenges. NH_3_ sensors operating in the subppm range (typical of near-emission source concentrations) are relatively new to the market, and while promising, there are metrological and technical challenges, especially in outdoor environments. Six NH_3_ sensors were evaluated under field conditions at a poultry house emission simulation site, Whim Bog, Scotland (3.4 g of NH_3_ min^–1^). Five electrochemical (TB600B, PS1, EC_tox_, duplicated AM1 low concentration (LC), and AM2 high concentration (HC)) and one chemiresistive-polymer (MELBA) sensors were tested and compared to a cavity ring-down instrument (Picarro G2103 NH_3_ analyzer) as a reference method. Only the TB600B (*R* ^2^ = 0.59–0.84), AM1 LC (*R* ^2^ = 0.7–0.9), and AM2 HC (*R* ^2^ = 0.71–0.9) demonstrated a positive correlation to the reference analyzer, being potentially capable of delivering indicative NH_3_ concentrations, with the caveat that the AM sensors had very low data capture and their performance may improve once the sensors operate for longer. All sensors tested have major technical challenges including accuracy, precision, response time, manufacturer deployment guidelines, sensor lifecycle metrics, software engineering, and data traceability. This study highlights the need for improvement in the NH_3_ sensor industry and among suppliers, and concludes that outdoor ammonia sensor measurements are not yet ready for routine use.

## Graphical abstract

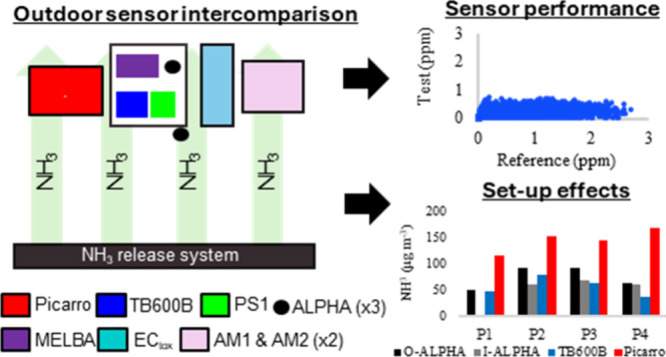

## 1 Introduction

Ammonia (NH_3_) is the major alkaline gas in the atmosphere.^1^ Although there are natural emissions, agriculture and particularly animal husbandry and fertilizer management emissions account for ∼90% of the total global emissions.^2,3^ Other emissions include traffic, refrigerants, and industry.^4–6^ Ammonia has also increasingly been used as an energy carrier and shipping fuel; therefore, new emission sources are likely to gain importance in the future.^7,8^ Policy makers have identified NH_3_ as a priority pollutant to mitigate^9^ due to the impacts to human health as one of the main drivers for PM_2.5_ formation,^10,11^ associated with several adverse health conditions.^12,13^

Accurate and precise measurements of NH_3_ are essential for both low-concentration environments^14,15^ (e.g., ecosystem protection; critical levels over sensitive ecosystems are set to 1–3 μg m^–3^) and near-source high-concentration environments^16,17^ (e.g., quantification of emission sources), as NH_3_ excessive deposition provokes acidification and eutrophication, leading to biodiversity losses.^14,18^ Measuring NH_3_ is challenging due to its physicochemical properties: semivolatile, highly reactive, and hydrophilic. Surface ambient atmospheric concentrations have high spatial and temporal variability as NH_3_ deposits readily to the surface, interacts with gasses to be taken up onto aerosol and water droplets,^19,20^ and has the potential to be re-emitted^21^ through volatilization processes.

Ammonia measurements are mostly done using diffusive sampling methods with offline chemical analysis.^20,22,23^ These methods have available published standards for diffusive sampling of ammonia in ambient air. Diffusive samplers are low-cost and typically used to provide exposure data ranging from one week or less to a full month^24^, reporting average concentrations over the integrated exposure period.^22,25^ There are a substantial number of automatic analyzers on the market, which generally require significant investment and expert users to ensure quantitative accurate measurements.^19,26–28^ There is no reference method for the automatic NH_3_ measurement.^26^

Theoretically, sensors combine the affordability and spatial flexibility of passive samplers with the high temporal resolution of automatic analyzers. Most NH_3_ sensors on the market are electrochemical (EC), where an electrolyte within the sensor reacts with the target gas producing an electrical signal proportional to the target gas concentration.^28,29^ Technical specifications of these sensors indicate that they are able to have good performance below 1 ppm of NH_3_; however, there is extremely limited evidence of the performance of these sensors in outdoor environmental conditions. EC sensor performance can be highly variable, with known challenges regarding accuracy and interferences from temperature and relative humidity (RH).^30–34^ These sensitivities make it challenging to monitor NH_3_ concentrations using EC sensors, both indoors and outdoors. Particularly critical are the potential cross-interferences of water molecules when quantifying NH_3_, as H_2_O molecules interact with the sensing surface.^35^

This paper represents, to the best of the author’s knowledge, the first outdoor ambient NH_3_ sensor intercomparison at a controlled NH_3_ release facility. We evaluated the performance of five commercially available NH_3_ sensors and an experimental chemiresistive sensor compared to an established high-time resolution NH_3_ analyzer under ambient conditions and both ambient level and high-concentration levels that are commonly observed near point sources. In addition to the analytical assessment challenges, the NH_3_ sensor market is difficult to navigate for nontechnical users and this study reports routes to manage these challenges associated with NH_3_ sensor calibration, setup, and deployment, reporting outcomes and recommendations for both sensor manufacturers and sensor users.

## 2 Materials and Methods

### 2.1 Sensor Selection

This study undertook market research on the commercially available NH_3_ sensors in the autumn of 2023 to select the most suitable options for outdoor monitoring. Based on technical and operational criteria (Table 1), five out of 15 (Table S1) commercially available EC NH_3_ sensors were selected: TB600B-NH_3_-10 (ECsense, Germany), EC_tox_-50-NH_3_ (ECsense, Germany), PS1–10-NH_3_ (SGX Sensortech, Switzerland), and the AM1 low-concentration (LC) and AM2 high-concentration (HC) sensors (Scentroid, Canada), designed to measure low and high NH_3_ concentration ranges, respectively. The MELBA sensor (IMT Nord Europe, France), based on chemiresistive detection,^36^ was added to the tested sensors as it complied with most of Table 1 requirements.

**Table 1:** Market Research Criteria Used to Select the NH_3_ Sensor Participating in the Whim Bog Campaign

criteria type	description of the specifications desired for the NH_3_ sensor
operational	the sensor needs to be <2 kg
	the sensor is installed in a device or control board that eases the user experience
	the sensor output is a digital signal
	the sensor needs to work under UK normal weather conditions (−10 to 30° and 50–100% RH)
technical	the sensor needs to have specificity toward NH_3_ in the range 0–20 ppm
	the LOD must be ≤1 ppm of NH_3_
	the resolution of the sensor must be ≤0.1 ppm
	the accuracy must be ≥5%
	the sensors lifetime is at least 12 months
	information present on cross-interferences occurring with other gaseous species
	the sensor adds other useful measurements such as T, RH, or other gaseous species

### 2.2 Field Site Description

Whim Bog is an ombrotrophic blanket bog located 11 km south of Edinburgh (NT 204532) 3°16′ W and 55° 46′ N. Whim Bog has been operated as an experimental nitrogen enhancement site since 2002, where ecosystem and species responses to N pollution (wet and dry deposited) are investigated. The site includes a dry NH_3_ gas enhancement transect^37–40^ with an ammonia plume dispersing over a distance of ∼100 m.

The NH_3_ enhancement field release system has previously been described.^37,38^ Ammonia is released when the wind blows at >2.5 m s^–1^ from the sector 180–215° for more than one minute. The release rate is set at 3.4 g NH_3_ min^–1^ from a 29 kg anhydrous NH_3_ cylinder with the flow rate fixed at 4.6 L min^–1^ determined by an Aera -FC7710C flow controller (maximum flow 10 L min^–1^ at STP). The gaseous NH_3_ passes along a 6 mm stainless steel tube before being injected into the airflow generated from a Wolter GMBH EK31 fan delivering ∼10 m^–3^ min^–1^. The diluted NH_3_ concentration is distributed from a 10 m line source (254 mm diameter pipe, with 4 mm holes perforated around and along the length of the pipe at 25 cm intervals), mounted 0.5 m above the vegetation. Annual average ammonia concentrations range from ∼70–100 μg m^–3^ (< 12 m) to ∼1–3 μg m^–3^ (ambient concentrations) (80–100 m) (Figure S1), with short-term peaks reaching very high concentrations up to >1000 μg m^–3^.

### 2.3 Reference Analyzer and Sensor Setup

The Picarro G2013 NH_3_ analyzer and sensors were installed 8 m away from the NH_3_ release system at Whim Bog (Figure 1a,b), in close proximity to the release point to ensure high concentrations (Figure S1). The experiment was carried out from 24/07/2024 to 28/08/2024. Both the Picarro analyzer and the sensors were placed 0.5 m above the boardwalk.

**Figure 1:**
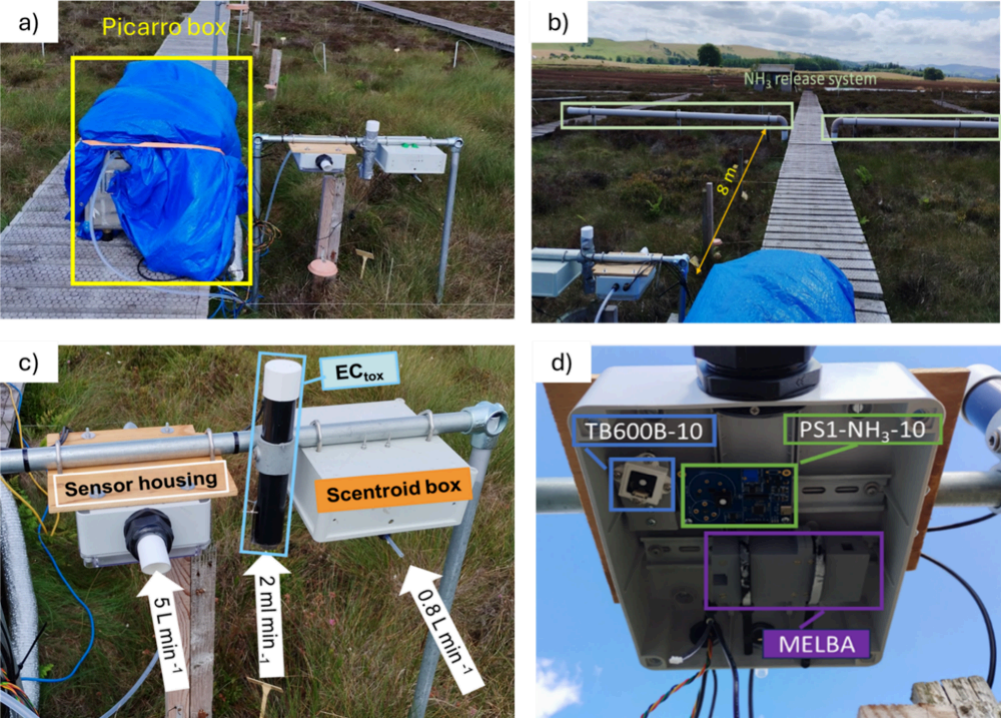
(a) Picarro box (left) and tested sensors (right). (b) Position of the NH_3_ release system from the sensor ensemble. (c) Disposition of the sensors in the sensor rack. (d) Sensors inside the sensor housing.

The Picarro analyzer was placed inside a weatherproof air-conditioned box with temperature control. The sensor housing (SH) was installed next to the Picarro box (Figure 1c). The SH was built for this experiment and consisted of a polycarbonate weatherproof box (24.5 cm × 20.5 cm × 9.5 cm) coated with PTFE to minimize surface absorption of water and NH_3_. The SH contained the PS1, TB600B, and MELBA sensors (Figure 1d). An external pump drew air at 5.5 L min^–1^ through a 30 cm PVC inlet allowing for the colocation of air sampling (residence time ∼ 52 s). From 19/8/24, 10 L min^–1^ flow was used to assess the influence of higher airflow (residence time ∼ 29 s). The EC_tox_ and Scentroid DR2000 units were placed next to the SH, sampling ambient air directly. The EC_tox_ was installed so its inlet probe initially oriented parallel to the ground; however; on 19/08/2024, it was rotated 45° toward the release system to enhance the exposure to the air stream released from the high NH_3_ emission system. The DR2000 unit was placed under a weatherproof box with a PFA inlet <10 cm and a 0.8 L min^–1^ flow (residence time ∼ 0.2 s).

### 2.4 Instrumentation

#### 2.4.1 NH_3_ Sensors

The technical specifications of the TB600B-NH_3_-10, EC_tox_-50-NH_3_, PS1–10-NH_3_, the AM1 LC and AM2 HC sensors, and the MELBA are summarized in Table 2. The AM1 LC and AM2 HC sensors were installed in duplicate in the Scentroid air quality monitoring product (DR2000). This unit also included particulate matter (PM) and carbon dioxide (CO_2_) sensors. All of the sensors have NH_3_ concentration ranges targeting between 0 and 10 ppm, with LODs between 0.01 and 3 ppm, and are designed to operate under “ambient conditions”, with most of them having optimal operational conditions of 20 °C and 50% RH.

**Table 2:** NH_3_ Sensor Details (Based on the Manufacturers' Technical Datasheets)^a^

sensor	detection	range (ppm)	LOD (ppm)	sensitivity (ppm)	time resolution(s)	response time (s)	airflow (l min^–1^)	other variables measured	price range (€)
TB600B-NH_3_-10	EC	0–10	0.01	0.01	60	3	n.a	T, RH	<150
PS1–10-NH_3_	EC	0–10	1	0.01	60	n.p	n.a	T, RH	<150
EC_tox_-50	EC	0–50	0.1	0.01	120 ^1^	60–600 ^1^	0.0002	T, RH,	650
AM1 LC	EC	0–10	0.03	0.001	60	40	0.8	T, RH, pressure, CO_2_; PM_*x*_	20,000 ^2^
AM2 HC	EC	0–100	3	1	60	50	0.2		
MELBA	chemiresistive	0–1	0.01	0.01	60	n.a	0.2	T, RH, fan speed	n.a.

a n.a: not applicable; n.p: not present; 1: depends on concentration; and 2: price refers to the DR2000R unit.

The PS1 and TB600B are 3-way electrode EC sensors attached to electronic boards that enable USB connectivity with a laptop running the supplier software for data recording and extraction (Figure S2). An external pump is required to pass air over the sensitive surface. The TB600B is a “ready to be used as soon as it is connected” sensor. The PS1 is sold as such, but it requires a two-point calibration before use (zero and span) to be carried out by the user. This was done in a laboratory chamber (0.28 l, polycarbonate) with N_2_ (99.998% purity, BOC) for the zero calibration and 100 ppm of NH_3_ (N_2_ 99,99%, NH_3_ 0.01%; BOC) diluted with N_2_ for span calibration to achieve 1 ppm NH_3_ (Figures S2 and S3). The supplier does calibrate the sensor in humid conditions; however, this was not possible to achieve due to laboratory setup limitations. The PS1 and TB600B sensors have an adjustable reporting time from one s up to one minute. The sampling period for the campaign was set to 1 min.

The EC_tox_ sensor consists of a 16 cm stainless steel probe with one end covered by a PTFE membrane that protects the sensing chamber. It samples air at 2 mL min^–1^ and analyzes for one minute. The EC_tox_ sensor used the calibration version of TB600B software to adjust the sampling time and record data.

The AM1 and AM2 sensors come as a part of the Scentroid DR2000R air lab product, a “black box” device equipped with T, RH, and seven atmospheric pollutant sensors designed to be installed into unmanned aerial vehicles for urban air quality surveys. It samples air at 0.8 L min^–1^ from the inlet located in front of the unit. Data collected by the DR2000R box are transmitted to a tablet-based ground station through wireless connection using a long-range protocol in real-time. The ground station is equipped with visualization and processing software to visualize and export the data; however, if the DR2000R connection is lost, data recording stops and needs to be restarted manually. The system battery and the ground station have a 2-h battery life, requiring a continuous power supply to enable extended monitoring.

#### 2.4.2 Picarro G2013 Analyzer

The Picarro G2013 NH_3_ Cavity ring-down spectroscopy (CRDS) analyzer uses infrared absorption.^19^ A 20 cm PFA inlet with a PTFE guard filter (0.001 μm porosity) sampled air at 1.5 L min^–1^ (residence time ∼ 0.22 s). The instrument's NH_3_ response (linearity and intercept) was checked in the laboratory before and after deployment (Table S2). A 100 ppm of the NH_3_ cylinder (N_2_ 99,99%, NH_3_ 0.01%; BOC) was diluted with N_2_ (99.998% purity, BOC) through mass flow controllers (MFCs) (Bronkhorst Ltd., Netherlands) (Figure S3). The Picarro prior to and after deployment demonstrated excellent linearity (*R* ^2^ = 0.999) across the whole concentration range (Figure S4). The slopes of the theoretical to measured concentrations were 0.90 (pre deployment) and 0.87 (post deployment). Prior to the deployment on Whim Bog, new guard PTFE filters (0.015 μm pore size, Entegris) were placed to protect the Picarro's analytical cavity. They were kept through the precampaign (no use) to the postcampaign (three months of use) linear check to assess if the exposure to high concentrations of NH_3_ during the intercomparison impacted the Picarro’s response. Picarro NH_3_ concentrations were not corrected to theoretical values due to potential losses of NH_3_ within the setup used to check the linearity of the instrument. For the purposes of testing the sensors, the Picarro was used as the “reference” with the assumption of a minimum of ± 15% uncertainty; however, previous tests^19^ show that the uncertainty lies within 5–7%. More information can be found in S4. The Picarro data's QA/QC involved checking the daily data streams and removing any data that would be out of the normal operational ranges of the temperature and pressure within the internal cavity.

#### 2.4.3 ALPHA Samplers

Adapted Low-cost Passive High Absorption diffusive samplers (UKCEH ALPHA)^22,23^ were deployed in triplicates on a weekly basis from 29/07/2024 until 28/08/2024 outside and inside the SH to estimate the average NH_3_ concentrations at each compartment and assess the potential gradient of NH_3_ concentrations. Table 3 shows the exposure periods and the amount of NH_3_ released at each one.

**Table 3:** Exposure Periods for the ALPHA Samplers during the Whim Bog Campaign^a^

period	ALPHA exposed	ALPHA removed	NH_3_ released (kg)
period 1	29/07/2024 16:27	05/08/2024 12:21	6.20
period 2	05/08/2024 12:35	12/08/2024 13:45	6.63
period 3	12/08/2024 13:45	19/08/2024 12:18	6.71
period 4	19/08/2024 12:32	24/08/2024 13:26	5.34

a Time is expressed as a local time.

#### 2.4.4 Data Analysis of Sensor Data against the Reference Instrument

The performance of the sensors during the campaign was assessed by checking the linear regression statistics of the 1, 15, and 60 min averaged data sets against the Picarro. As an additional evaluation, a set of statistical metrics^41^ were used to evaluate how well the sensors aligned with the reference. This approach is widely used to assess the performance and accuracy of dispersion pollutant models against real observations.,^42,43^ This study used this methodology to evaluate the Whim Bog sensors (considered the tested models) against the Picarro analyzer (used as the reference) and assess their performance in both ambient and high concentration environments. The metrics used are fractional bias (FB), geometric mean bias (MG), normalized mean square error (NMSE), geometric variance (VG), correlation coefficient (*R*), and fraction of predictions within a factor of 2 of observations (FAC2). Details on the equations can be found in the study by Chang and Hanna.^44^ For a perfect agreement between the test and reference data sets, the ideal values are 1.0 for FAC2, MG, and VG and 0.0 for NMSE and FB. FB and MG measure mean relative bias, capturing only systematic errors, whereas NMSE and VG assess mean relative scatter, reflecting both systematic and random errors.

## 3 Results

### 3.1 Overview of the Campaign

Figure 2 presents the meteorological conditions during the campaign. The temperatures ranged from 13.6 ± 3 °C and humidity ranged from 81.2 ± 12.8%. While these conditions are within the typical range for Scotland, they are significantly different from the test conditions reported by the manufacturers (≈20 °C, 50% RH). The mean wind speed was 4.3 ± 2.2 m s^–1^, with wind direction predominantly from the SW–SE, with occasional N–NE. The NH_3_ release system was on ∼18% of the time during the campaign. This resulted in both high and low NH_3_ concentrations. Sporadic rainfall events occurred throughout the campaign, particularly during the periods 4–10th August and 12–7th August.

**Figure 2:**
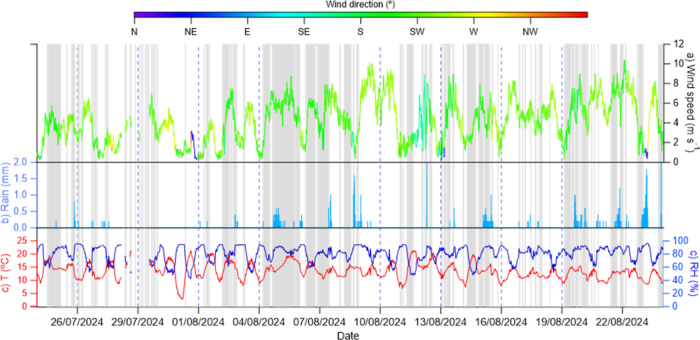
Time series of meteorological conditions during the campaign (top) including (a) wind speed and direction, (b) precipitation, and (c) air temperature and RH at the field Whim Bog for the period 24/07/2024 to 28/08/2024. Gray bars in the top panel are when the NH_3_ release system was activated, releasing NH_3_ at a rate of 3.4 g NH_3_ min^–1.^.

Figure 3 shows the reported NH_3_ concentration time series alongside the release system activation. Data capture (DC) was variable, with the Picarro analyzer (99.7%), PS1 (88.2%), TB600B and EC_tox_ (68.4%), and AM1 LC and AM2 HC sensors (5.6% each). Two periods of system downtime (first period: 02/08/2024 05:13–02/08/2024 15:44; second period: 11/08/2024 23:08–12/08/2024 00:07) were excluded from the statistical analysis. The data losses from the TB600B and EC_tox_ were caused by software crashes and malfunctions. The low DC from the AM sensors was due to the DR2000R unit software shutting down the data recording after 6 h of operation, requiring a manual restart after the shutdown of both the data recording software and the DR2000R unit; this prevented using an automatic restart system, leading to unavoidable data losses. The MELBA sensor worked only for a couple of hours of exposure and then saturated after 12 h of exposure. The MELBA was therefore excluded from further inclusion in the study. The data obtained is shown in S4 of the Supporting Materials.

**Figure 3:**
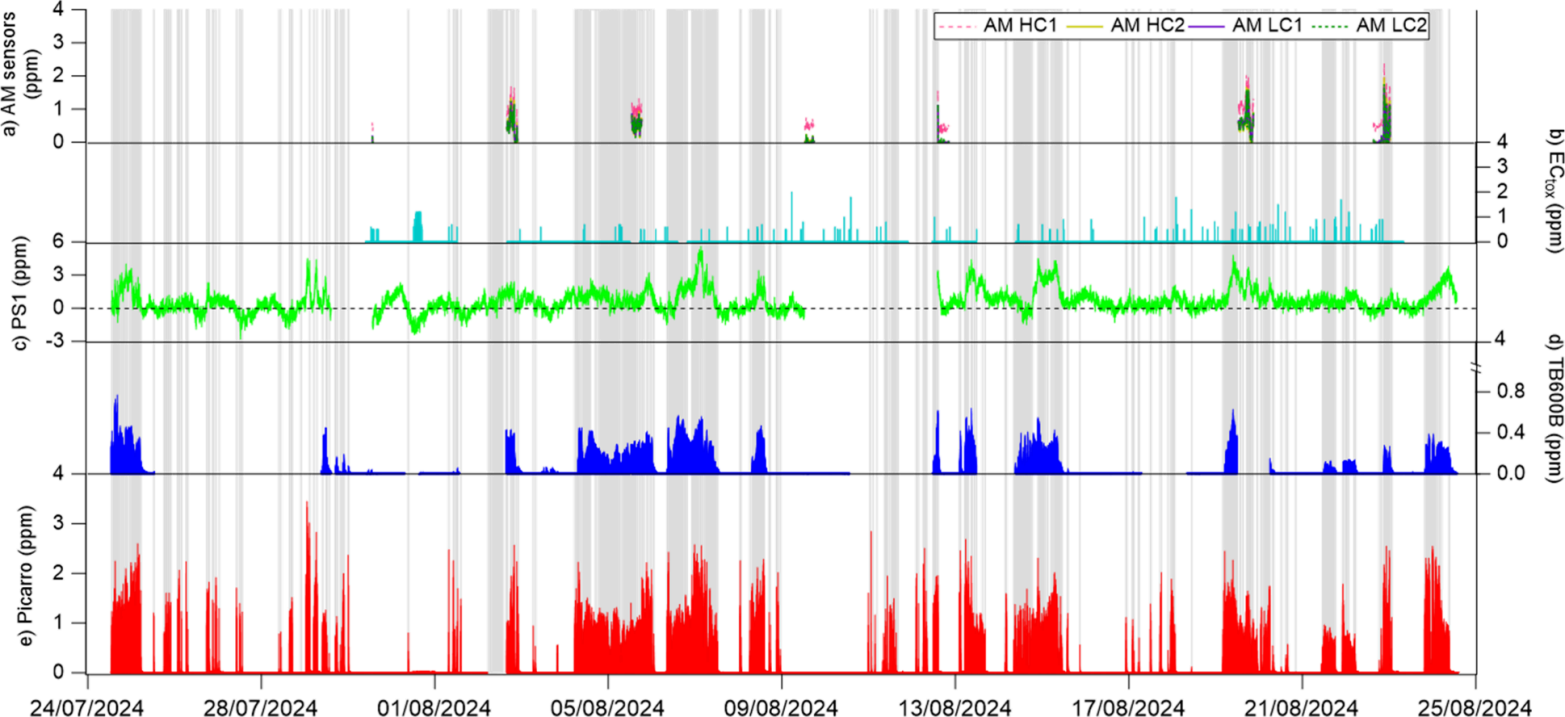
Time series of NH_3_ concentrations reported by the CRD and sensors during the Whim Bog campaign for the period from 24/07/2024 to 28/08/2024. From top to bottom: (a) AM sensors, (b) ECtox, (c) PS1, (d) TB600B, and (e) Picarro analyzer. The dashed line in the PS1 sensor corresponds to the 0-ppm value. Gray bars indicate the NH_3_ release system being on.

The reported NH_3_ concentrations by the Picarro ranged between 0.001 and 3.5 ppm of NH_3_, covering ambient background through to near-source concentrations. The Picarro responded rapidly (<1 min) to concentration changes between the release and nonrelease periods (Figure 3). The sensor responses when the release system started were highly variable: The TB600B reported higher NH_3_ concentrations when the release system was active, although the absolute concentration remained below 1 ppm and did not always follow the same temporal patterns as the Picarro. The PS1 output was highly variable with readings between −3 and 6.5 ppm of NH_3_. The higher concentrations recorded corresponded to the NH_3_ release (e.g., during the evening of 06/08/2024, when >4 ppm of NH_3_ was observed), but the signal became unstable and noisy as NH_3_ concentration returned to ambient values. The EC_tox_ reported 0 ppm for most of the campaign and was unresponsive to high concentrations during release periods; instead the sensor reported high concentrations randomly in the output, such as those observed on 09/08/2024. The low DC of the AM sensors made it difficult to assess their performance; however, it is noted that the data from those operational periods showed a fast response to increases in NH_3_.

### 3.2 Statistical Analysis between Sensors and the Reference

#### 3.2.1 Linear Regressions between Picarro and Test Sensors

The performance of the sensors against the reference was assessed by plotting the data sets at 1-, 15-, and 60 min averages. The linear regression fit and correlation statistics calculated are shown in Figures 4, 5, and Table 4. The EC_tox_ did not demonstrate a relationship with the reported concentrations from the Picarro for all signal averages periods; therefore, no statistics are shown. The AM1 LC and AM2 HC sensors had the best correlation statistics with good slopes (AM1 LC1: 0.68, 0.83, 0.85; AM1 LC2: 0.68, 0.82, 0.83; AM2 HC1: 0.7, 0,84, 0.84; AM2 HC2: 0.72, 0.87, 0.87) and the best *R* ^2^ out of all sensors (AM1 LC1: 0.7, 0.89, 0.9; AM1 LC2: 0.7, 0.88, 0.9; AM2 HC1: 0.71, 0.88, 0.88; AM2 HC2: 0.72, 0.9, 0.9); however, their very low DC (5.6%) is too low for meaningful statistical validation or as a representation of the sensor’s capabilities. The slopes of the TB600B (0.23, 0.3, 0.32) and PS1 (1.41, 1.87, 1.99) show an underestimation and overestimation of NH_3_ concentrations, respectively, although the *R* ^2^ coefficients are quite satisfactory for the TB600B (0.59, 0.77, 0.84).

**Table 4:** Correlation Parameters for the Linear Regressions between the Picarro and Tested Sensors^a^

		time average (min)		
sensor	parameter	1	15	60
TB600B	slope	0.23	0.3	0.32
	offset	0.03	0.02	0.01
	*R* ^2^	0.59	0.77	**0.84**
	N data	30,349	2034	515
PS1	slope	1.41	1.78	1.99
	offset	0.32	0.23	0.19
	*R* ^2^	0.33	0.44	0.5
	N data	38,243	2551	641
AM1 LC1	slope	0.68	**0.83**	**0.85**
	offset	0.06	0.01	0
	*R* ^2^	0.7	**0.89**	**0.9**
	N data	2449	170	47
AM1 LC2	slope	0.68	**0.82**	**0.83**
	offset	0.06	0	0
	*R* ^2^	0.7	**0.88**	**0.9**
	N data	2449	170	47
AM2 HC1	slope	0.7	**0.84**	**0.84**
	offset	0.5	0.45	0.44
	*R* ^2^	0.71	**0.88**	**0.88**
	N data	2449	170	47
AM2 HC2	slope	0.72	**0.87**	**0.87**
	offset	0.05	–0.01	–0.01
	*R* ^2^	0.72	**0.9**	**0.9**
	N data	2449	170	47

a Slopes between 0.8 and 1.2 and *R* ^2^ values above 0.8 are highlighted in bold.

**Figure 4:**
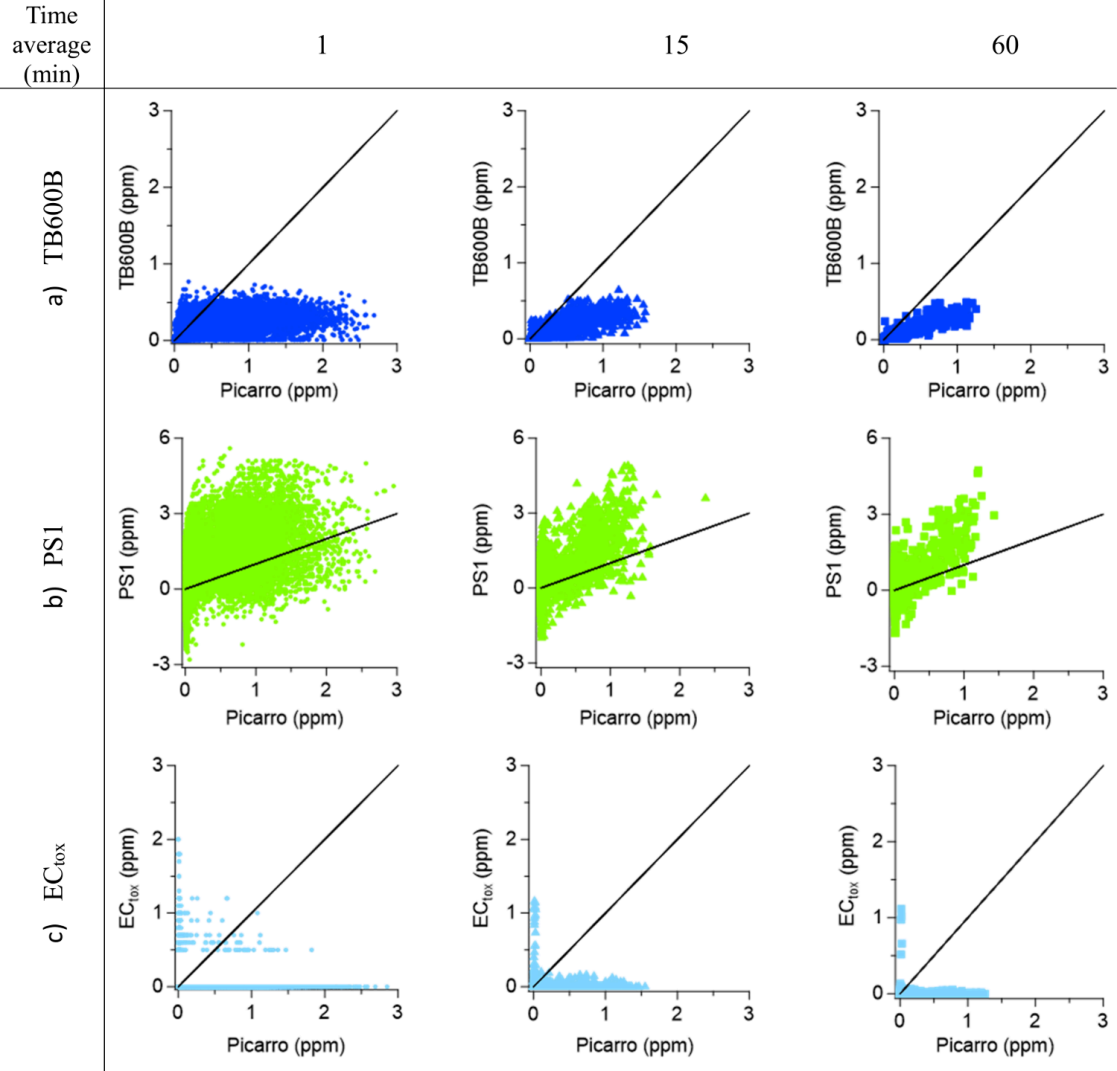
Linear regression plots between the Picarro analyzer's (*x* axis) and sensors' (*y* axis) NH_3_ concentrations (expressed in ppm) during the Whim Bog campaign at 1-, 15-, and 60 min time resolution for the TB600B (a), PS1 (b), and EC_tox_ (c). The black lines on the graphs represent the 1:1 line.

**Figure 5:**
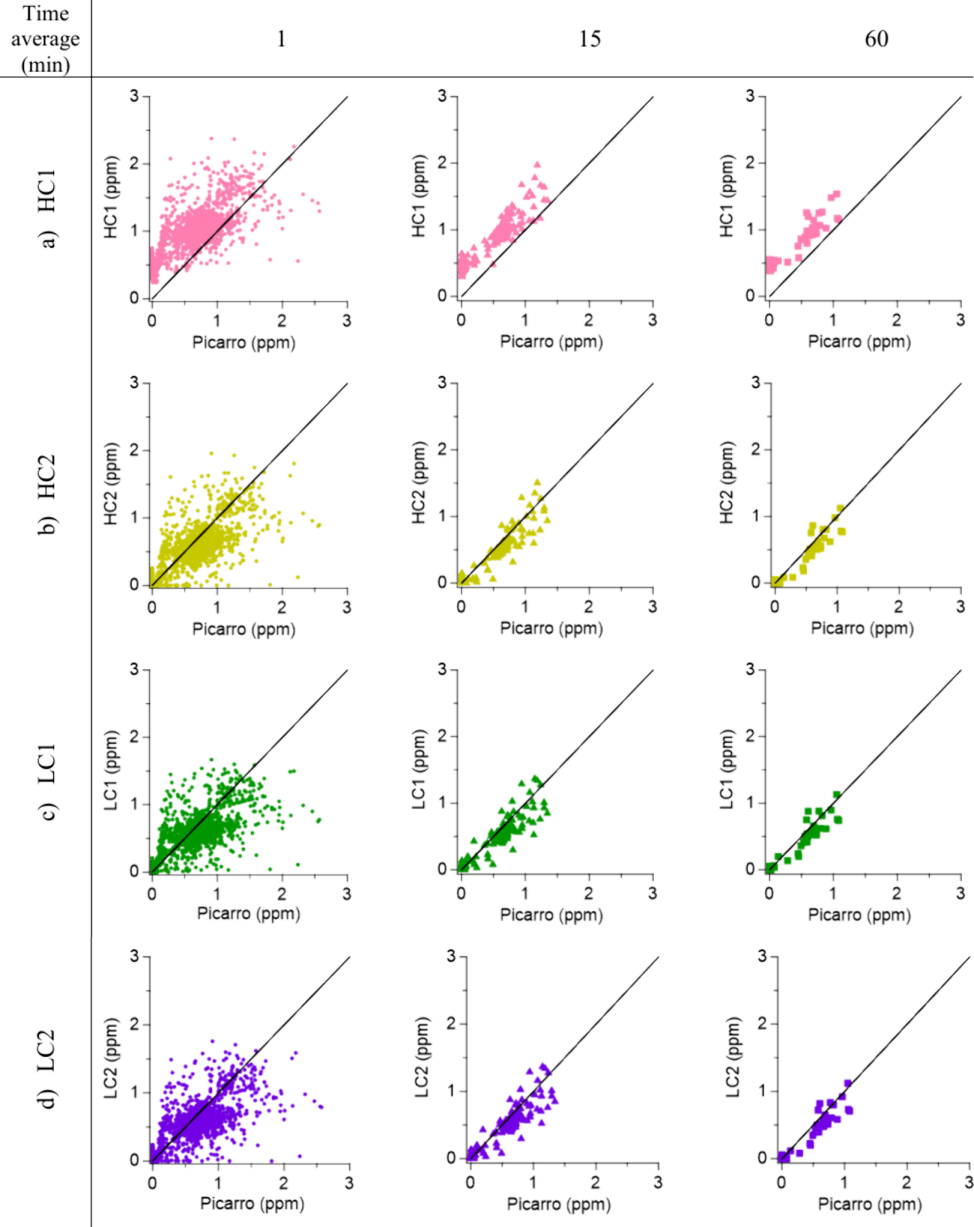
Linear regression plots between the Picarro analyzer's (*x* axis) and sensors' (*y* axis) NH_3_ concentrations (expressed in ppm) during the Whim Bog campaign at 1-, 15-, and 60 min time resolution for the AM1 HC1 (a), AM1 HC2 (b), AM2 LC1 (c), and AM2 LC2 (d). The black lines on the graphs represent the 1:1 line.

The sensors inside the SH show distinctive behaviors. The TB600B 1 min time resolution data set has a low slope (0.23) but a moderate *R* ^2^ (0.59). The 1-h data set improves the regression parameters (slope = 0.32; *R* ^2^ = 0.84); however, the sensor consistently underestimates NH_3_ concentrations by a factor of 4–5 times. The PS1 sensor exhibits high variability in its concentration range, from −3 to 2 ppm of NH_3_ in ambient air up to 6.5 ppm of NH_3_ when the release system was on. The EC_tox_ showed no correlation with the reference, as the higher concentration peaks occurred during periods when no NH_3_ was being released (Figure 4). The AM1 LC and AM2 HC sensors were present as duplicates in the DR2000. Figure S6 shows that the correlation plots between the AM2 HC sensors are similar; however, HC1 had an offset of 0.45 ppm of NH_3_ (Table S3). The AM LC sensors behave similarly both among themselves (slope = 0.99, *R* ^2^ = 0.97) and against the Picarro analyzer.

From this study, only the TB600B would be considered potentially suitable for ambient outdoor monitoring purposes in the “out of the box” application; however, further testing would be required to understand the baseline and range of the sensor. The AM1 LC and AM2 HC showed promising results, but more data is required to understand the true capabilities of the AM sensors.

#### 3.2.2 Evaluation of the Sensors against the Reference

The results of the performance evaluation of the tested sensors against Picarro are shown in Table 5. The 15 min data set was used for this comparison as a compromise between the smoothing of the sensors' data and the available number of data points used for the comparison.

**Table 5:** Performance Metrics for the 15 min Resolution Dataset between the Test Sensors and the Picarro Analyzer^a^

sensor	FB (−0.3 < FB < 0.3)	MG (0.7 < MG < 1.3)	NMSE (<1.5)	VG (VG < 4)	*R* (%) (>0.5)	FAC2 (%) (>0.5)	# data	DC (%)
TB600B	0.95	1.81	**0.61**	**3.16**	**0.86**	**0.53**	1512	68.87
PS1	–0.99	0.14	**–1.42**	687.51	**0.66**	0.22	1517	88.18
ECtox	**–0.05**	0.42	**–25.10**	428.21	–0.29	0.11	112	68.41
HC1	–0.53	0.27	**–0.41**	31.98	**0.93**	**0.62**	132	5.6
HC2	**0.17**	1.35	**0.31**	**1.42**	**0.88**	**0.89**	97	5.6
LC1	**0.15**	**1.13**	**0.38**	**1.24**	**0.89**	**0.89**	112	5.6
LC2	**0.20**	**1.29**	**0.34**	**1.55**	**0.88**	**0.82**	107	5.6

a Below the header of each metric is shown the acceptance range. Sensors within the acceptance threshold are highlighted in bold. A perfect model would have MG, VG, R, and FAC2 = 1.0 and FB and NMSE = 0.0.

All measurements are subject to uncertainties, and the performance metrics selected are inherently nonexhaustive. Hanna and Chang^45^ proposed that even an acceptable model may not meet all acceptability criteria for all experiments and thus set that at least half of the performance tests should be successfully passed in order to consider a test sample comparable to the reference.

All sensors had at least two of the six metrics within the acceptable range. The lowest performing sensors were EC_tox_ and the PS1. The EC_tox_ passed the FB (−0.05) and NMSE (−25.1) metrics accounting for systematic bias and random scatter; however the EC_tox_ recorded zero concentrations for most of the campaign; when responded, it appeared to be independent of the NH_3_ release system, which explains its poor statistical performance overall. The other sensor with deficient performance was the PS1, only passing the NMSE (−1.42) and *R* (0.66) metrics; the latter was the lowest value among all accepted sensors. The PS1 sensor had issues stabilizing at ambient concentration levels, fluctuating between −2.5 and 3 ppm of NH_3_. This indicates that the sensor performance is affected by outdoor environmental conditions and probably noise is dominating the signal.

The TB600B passed the NMSE (0.61), VG (3.16), *R* (0.86), and FAC2 (0.53) metrics, failing the FB and MG, which mainly account for systematic bias. This is not surprising, as the FB is influenced by high outliers and the MG by near-zero concentration values. The TB600B consistently reacted to the high NH_3_ emission periods, yet consistently underestimated the reference NH_3_ concentrations by ∼75% and was below the sensor detection limit periods when the NH_3_ was not being released (from manufacturer specifications). Although the Picarro and TB600B have a degree of agreement, these metrics fail and are thought to be from analytical capabilities of each technique and a calibration/scaling issue with the TB600B.

The AM1 LC and AM2 HC sensors had variable performance. The LC series performed better than the HC ones, as the HC1 sensor only passed NMSE (−0.41), *R* (0.93), and FAC2 (0.62), while the HC2 sensor passed all metrics except MG (1.35). Even though both sensors fail the MG metric, they do fall on the opposite sides of the accepted value range; the HC1 sensor has a systematic offset of 0.44 ppm, likely causing the disagreement between the Picarro and AM sensor for FB, MG and VG, while the HC2 scores were almost within the accepted range for MG, indicating an overall better performance of the system when the offset is not present. The LC series achieved all metrics within the acceptable range, indicating the overall best performance for the available data. Although the AM LC sensors demonstrated the best performance among all of the sensors, the low DC limits the applicability of the Chang and Hanna tests.

#### 3.2.3 Response Time Tests

NH_3_ concentrations have high temporal variability in outdoor environments, making response time one of the most critical parameters to consider when selecting a sensor. Following the field intercomparison, a short laboratory test of the response of the TB600B was carried out in a test chamber previously used to calibrate the PS1 (Figure S2) to assess its performance while conducting the postcampaign linear check of the Picarro (Figure S4). The TB600B was placed <5 cm from the inlet tube with the sensitive element oriented perpendicularly to the inlet. Figure 6 shows the time series for the reported NH_3_ concentrations for Picarro and TB600B.

**Figure 6:**
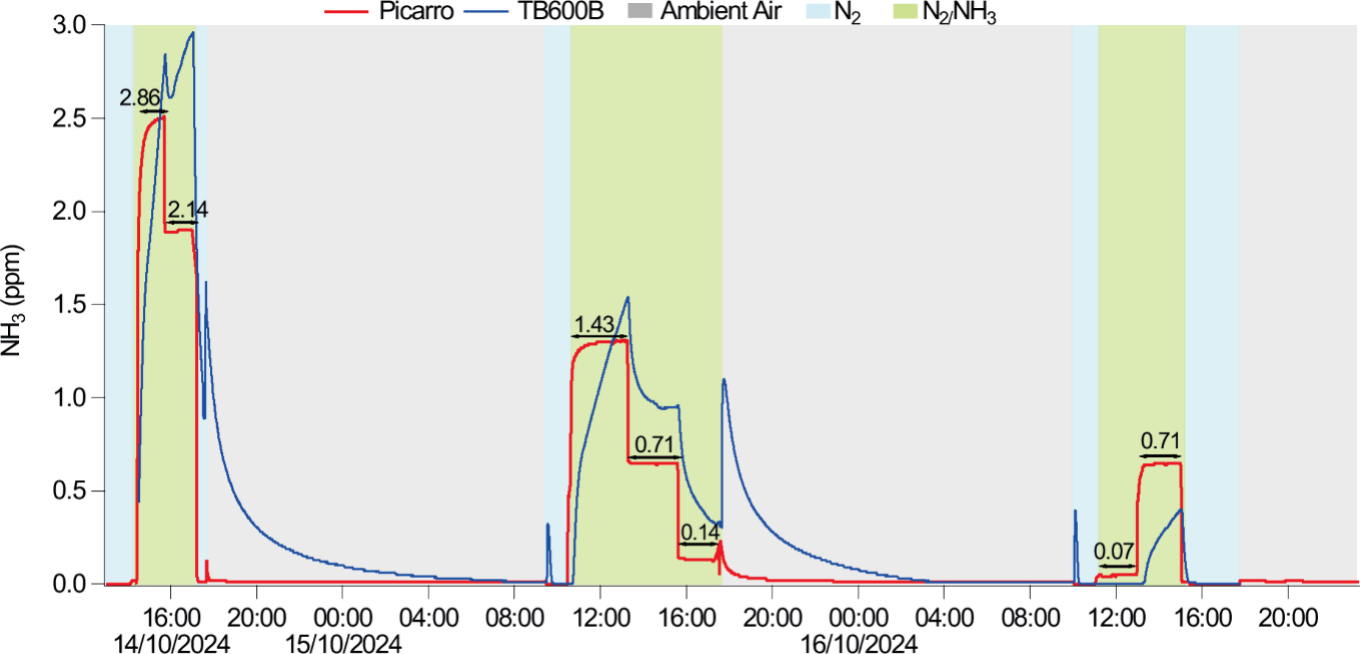
Time series of NH_3_ concentration recorded by the Picarro and TB600B instrument during the test chamber experiment. The colored backgrounds indicate whether the sensors were exposed to ambient air (gray), pure N_2_ (blue), or NH_3_/N_2_ mixtures (green). The arrows and concentrations above the Picarro plateau stages correspond to the theoretical NH_3_ concentration values in ppm.

The Picarro quickly responded to concentration changes between N_2_, the N_2_/NH_3_ mixture, and ambient air. This was particularly evident during the ascent periods when it plateaued after ∼30 min. The TB600B consistently showed slower responses and did not stabilize at any concentration level. This responsiveness lag was most pronounced after NH_3_ exposure, where the TB600B took ∼11 h to decrease from the maximum concentration level to ambient air concentrations. Similar patterns were observed when testing the TB600B between 1 and 2 ppm of NH_3_; however, it was impractical to assess whether extended sampling times would have resulted in achieving a concentration plateau. Notably, the Picarro followed stepwise decreases from theoretical 2.87 ppm of NH_3_ down to 2.14 ppm of NH_3_, whereas during this phase, the TB600B increased its concentration reading to 2.97 ppm of NH_3_. It is unclear whether this was caused by the slow response time of the sensor reacting to the previous concentration level or due to a small pressure change caused by the changes in N_2_/NH_3_ mixtures from the MFCs. Small concentration spikes were observed by the TB600B as the flow settings were changed, which may indicate that the sensor electronics are susceptible to sudden pressure and RH changes; however, these were not captured by the Picarro. The TB600 suppliers do not recommend using the sensor below 40% RH; therefore, these results may not be representative of normal performance. This restriction raises challenges in performance testing with traceable NH_3_/H_2_O/N_2_ mixtures and further reinforces the necessity for suppliers and the metrological community to develop methodologies that allow testing these types of sensors without compromising their effective life use.

#### 3.2.4 Sensor Housing Effects over Sensor Measurements

To assess if NH_3_ concentrations inside the SH were different from those outside the SH, ALPHA samplers were deployed both outside (O-ALPHA) and inside (I-ALPHA) during the four periods. Figure 7 shows the average ALPHA NH_3_ measurements compared to the averaged Picarro and TB600B-NH_3_ concentrations during the ALPHA exposure periods (Table 3).

**Figure 7:**
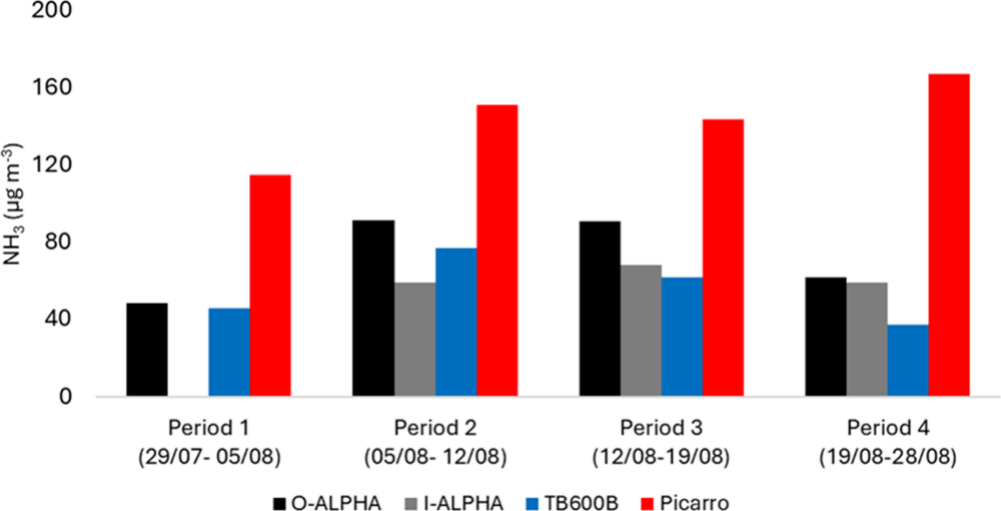
Average NH_3_ concentrations for O-ALPHA, I-ALPHA, TB600B, and Picarro for the ALPHA exposure periods.

The Picarro recorded the highest NH_3_, as it captured NH_3_ spikes unresolved by ALPHA and TB600B (Table S5). The O-ALPHA measured concentrations ≈60% lower than the Picarro, except for Period 1 (40%; Table S6), likely because O-ALPHA was particularly wet during Period 1; excessive moisture in an ALPHA sampler can lead to reactive coating loss. Period 1 I-ALPHA samples were discarded as they were soaked upon retrieval.

On average, O-ALPHA measured 21% higher NH_3_ concentrations than I-ALPHA except during Period 4, where differences narrowed to 4%. This change may be attributed to the increased airflow (5.5–10 L min^–1^), enhancing diffusion within the SH and reducing the internal concentration gradient. However, this contrasts with the TB600B-Picarro comparison: during Period 4, the TB600B underestimated NH_3_ concentrations by a factor of 4.45, compared to a factor of 2.3 observed during Periods 1–3. These inconsistencies are likely caused by the sampling setup and analytical differences, limiting the conclusions on the relationships between the sampling rate, NH_3_ concentrations measured, and sensor sensitivity.

Overall, the results indicate that the SH impacted measured NH_3_ concentrations, highlighting the need for optimized sensor setups. Further research is required to properly characterize SH effects for effective sensor deployment strategies for outdoor monitoring purposes.

## 4 Discussion

### 4.1 Sensor Performance

This study has tested the performance of commercially available NH_3_ sensors under outdoor conditions at concentrations near NH_3_ emission sources. None of the sensors had quantitative (±15%) or even qualitative performance (±30%) compared to the reference. Sensor response times for both concentration increases and decreases were highly variable and in general significantly slower than NH_3_ concentration fluctuations (Figure 6). The EC_tox_ worked for more than half of the campaign, but its response did not correlate with the NH_3_ emission source. The PS1 operated continuously throughout the intercomparison, yet it did not correlate with the Picarro’s NH_3_ concentration. The AM1 LC and AM2 HC sensors had the best linear regressions and statistical performance metrics for the short periods they were operational, but they cannot be taken as representative of their performance due to the very low DC (5.6%).

The TB600 passed 4 out of 6 performance metrics and worked for more than half of the intercomparison, indicating a good degree of agreement with the Picarro and providing a representative assessment of its capabilities. The TB600 signal responds in proportion to the NH_3_ release; however it does not measure the expected/theoretical concentrations. Field concentrations were underestimated consistently, while the laboratory testing overestimated them, hence the TB600 did not reach the qualitative or quantitative standard. The sensor has a fast and a slow response component: with a rapid response to changing NH_3_ concentration but a slow response component to get a stable reading. This indicates that accurate concentration measurements with the TB600 in a fluctuating NH_3_ concentration field are not possible; however, indicative relative changes of NH_3_ concentrations are recorded. In theory, as long as the peak concentration is proportional to the accurate concentration and this does not change as a function of time when the sensor is operating, this characteristic could serve as a useful response function, with regular calibration. However, the TB600B outdoor applications are very limited by the systematic underestimation of near-source NH_3_ concentrations by a factor of two to four, and improvements on the sensor technology and SH are needed in future developments to decrease this bias.

One concern when placing the sensors inside the SH is the NH_3_ concentration gradient between outdoors and indoors (Section 3.2.4). The SH is necessary for outdoor operation, protecting sensors from hazards such as rain, dust, or insects. However, NH_3_ adsorption onto internal SH surfaces likely reduces internal concentrations compared with outdoor levels, but this should be systematic over time. Sensor developers must take into consideration this gradient when designing the SH for the next generation of sensors. Laboratory tests^46^ showed that better performing sensors feature large effective surface areas, high airflow, and a perpendicular (90°) orientation to the analyte stream. To prevent potential outdoor hazards, sensors in this study were mounted facing downward (180°) in parallel to the airstream.

Although T and RH may influence sensor performance and should be isolated in the laboratory from other variables present in outdoor environments, their influence was estimated via linear regression against the measured NH_3_ concentrations when the release system was on, as ambient NH_3_ levels were below the sensor’s LODs. Table 6 and Table 7 show the linear regression statistics for T and RH, respectively.

**Table 6:** Linear Regression Parameters of T against NH_3_ Concentrations (1 min) for the Different Sensors while the NH_3_ Release System Was On

sensor	slope ± error	offset ± error	N data	*R* ^2^
Picarro	1.28 ± 0.03	–0.03 ± 0.001	7689	0.04
TB600B	0.14 ± 0.01	0.01 ± 0.001	6416	0.03
PS1	3.05 ± 0.07	–0.1 ± 0.01	7836	0.05
EC_tox_	0.003 ± 0.01	0.0002 ± 0.0004	5709	2.99 × 10^–5^
AM HC1	1.94 ± 0.04	–0.05 ± 0.003	1019	0.26
AM HC2	1.28 ± 0.05	–0.04 ± 0.003	1019	0.14
AM LC1	1.21 ± 0.04	–0.03 ± 0.003	1017	0.14
AM LC2	1.25 ± 0.04	–0.04 ± 0.003	1017	0.15

**Table 7:** Linear Regression Parameters of RH against NH_3_ Concentrations (1 min) for the Different Sensors while the NH_3_ Release System Was On

sensor	slope ± error	offset ± error	N data	*R* ^2^
Picarro	0.16 ± 0.04	0.01 ± 0.001	8072	0.03
TB600B	0.63 ± 0.01	–0.004 ± 0.0002	6416	0.11
PS1	0.81 ± 0.11	0.0001 ± 0.0001	7836	0.01
EC_tox_	–0.05 ± 0.01	0.0001 ± 0.001	5709	0.0001
AM HC1	–0.5 ± 0.13	0.02 ± 0.002	1019	0.13
AM HC2	–0.81 ± 0.14	0.02 ± 0.002	1019	0.10
AM LC1	–0.69 ± 0.12	0.02 ± 0.001	1017	0.11
AM LC2	–0.75 ± 0.13	0.02 ± 0.002	1017	0.10

Very weak correlations (*R* ^2^ < 0.3) were observed between sensors, RH, and T. Positive trends were observed for TB600B with T (Figure S7b), RH, and AM sensors (Figure S10), while negative trends were observed for Picarro, PS1, and AM sensors with T (Figures S7a and S8). Table S4 shows that sensors were used out of the suppliers' expected ranges of RH (mostly due to rain events, Figure 2), and in the case of the MELBA, the NH_3_ range (Figure S5); however, future improvements need to address their performance under harsh outdoor conditions.

According to suppliers, most sensors correct the raw signal with T and RH through proprietary algorithms. Assessing T and RH biases in outdoor conditions is challenging due to the high variability of NH_3_ concentrations, influenced by the release system, the atmospheric dispersion, and deposition and remission processes. Further laboratory testing and greater transparency from suppliers would help optimize sensor selection for specific applications.

It is beyond the scope of this study to establish clear relationships between the sampling rate, NH_3_ concentrations measured, and changes in sensitivity of the sensor as this intercomparison aim was to test basic NH_3_ sensor performance in outdoor conditions. The airflow change from 5.5 to 10 L min^–1^ in the SH did not change the TB600B performance compared to the Picaro (Figure S11), likely due to the uneven amount of data across periods; therefore, definitive conclusions cannot be extracted. NH_3_ sensor manufacturers and distributors should provide more detailed information regarding the sampling method and airflow. The physical setup is as important as sensor capability for outdoor measurements of NH_3_, as previously noted for the automatic NH_3_ analyzers.^19^

### 4.2 Available Information for the End User

One of the main challenges when starting to use NH_3_ sensors, despite the variety on the market, is the lack of information provided by sellers. Topics such as detailed information on the sensor working principle, data collection, and how to install and operate them to obtain meaningful measurements are not covered. In the market research the authors undertook in planning the intercomparison, the technical terminology was not consistent across manufacturer/seller technical sheets. Operational time resolution, limits of detection, response time, and cross interference lists were missing for some sensors.

Calibration certificates were provided for the TB600B and AM sensors by the manufacturers. The TB600B had a manufacturer 2-point calibration (0 and 8 ppm of NH_3_), while the AM sensors had a 3-point calibration: the HC sensors were documented as tested for 24, 10, and 5 ppm of NH_3_, while the LC sensors were tested for 10, 5, and 1 ppm of NH_3._ These concentration levels are within the expected range for indoor agricultural and industrial uses; however, they are far from the theoretical LOD reported in the datasheets (Table 2). Furthermore, the TB600B, AM1 LC, and AM2 HC sensors were tested under similar environmental conditions (22.5–23 °C and 45–50% RH), which are applicable to indoor environments. However, the AM sensors are part of an outdoor air quality monitoring product, which should be designed for ambient RH values (typically 60–99%) and ambient temperatures (e.g., in Scotland −10 to 30 °C). The technical sheets record the change of sensitivity in nA per ppm of NH_3_ at a specific temperature. The issue of RH affecting response is also an issue for nonwet chemistry based NH_3_ analyzers^47^ and should be reported in the technical specifications.

The TB600B and Scentroid AM1 and AM2 systems were ready to be used as they arrived from the supplier, albeit with limited information on how to setup and run, and only the Scentroid DR came with a user guide to start the measurements. The EC_tox_ and PS1 sensors required some degree of laboratory preparation that was not specified in the technical sheet. This was a particular issue for the PS1, as it requires calibration by the user prior to deployment, either by using a gas hood purchased separately from the manufacturer or directly exposing the sensor to controlled concentrations of NH_3_. The EC_tox_ sensor has an integral pump and a sensing chamber inside its housing; however, as no information on the working principle was available, supplier support was needed to get the sensor working and, later in the campaign, to change the sampling time to be comparable to other sensors.

Understanding cross-interferences from other gaseous species is important for using EC sensors; however, even though all of the sensors are EC-based, different cross-interferences were recorded in their technical sheets. SO_2_ and CO are listed as cross-interferences for the PS1 and EC_tox_; however, the direction and magnitude of the interference varied between sensors. This could be understandable if the 3-way electrode sensors were different; however, conversations with some of the suppliers revealed that all of the 3-way electrode sensors came from the same manufacturer, with sensor suppliers implementing different signal processing, physical protocols, software visualization, or integration. Some of these cross-interferences could be attenuated by establishing regular comparisons between standardized analyzers and sensors, a common practice when using low-cost sensors as their data sets can be checked and correction factors could be applied if the comparison highlights potential cross-interferences and sensor drift.^48^ Regulated pollutants such as PM_*x*_ and NO_*x*_ have standardized high-temporal-resolution methods for their measurement^9,49,50^ or even standardized performance evaluation methods for portable sensors.^51^ In contrast, passive diffusive samplers remain the only standardized method for NH_3_,^52^ limiting the high-time-resolution capabilities of NH_3_ sensors. Future improvements in high-time-resolution NH_3_ methods will allow for the correction and calibration of these sensors.

## 5 Conclusions

This study compared the performance of six sensors, TB600B, PS1, AM series, MELBA, and EC_tox_, against a high-time-resolution Picarro NH_3_ analyzer under outdoor conditions at an experimental NH_3_ enhancement field site. Results demonstrated that NH_3_ sensors are not ready to be used without expert assessment and even then do not provide more than indicative NH_3_ concentrations.

The TB600B and AM sensors show promise for near-source outdoor NH_3_ monitoring, with the caveats that the TB600B notably underestimated concentrations due to technical constraints and housing effects, while AM sensors displayed the best agreement with the reference analyzer, but suffered from a low DC. No significant T or RH effects were observed except for the MELBA sensor, which saturated at RH > 90% and NH_3_ > 1 ppm; caution is required not to over interpret these results, as the opacity of the sensors' data processing makes it challenging to assess the influence of environmental parameters in the field. Further laboratory characterization is needed to correct these biases and optimize sensor performance.

This study highlighted the need for the NH_3_ sensor industry suppliers to improve harmonization and for NH_3_ sensor users to be rigorous around using standardized testing protocols for calibrating, maintaining, and performing QA/QC on the sensor data sets. Future improvements of NH_3_ sensors should improve the sensitivity and response time to meet the need for monitoring NH_3_ high temporal variability.

## Supplementary Material



## Notes

Sensor selection process, the setup on Whim Bog, the linear checks carried for both the reference analyzer and NH_3_ sensors, the performance of the MELBA sensor, the intrasensor variability between AM sensors, the potential interferences of T and RH over sensor’s NH_3_ measurements, and the potential influence of changes in the setup over sensor’s NH_3_ measurements (PDF)
