# Supplementary material for: Are Ammonia Sensors Ready for Outdoor Use?
Source: ACS Omega. 2025 Dec 29;11(1):917–29. doi: 10.1021/acsomega.5c07873 (PMC12809547; doi:10.1021/acsomega.5c07873)
Supplement: Supplementary file 1 [file ao5c07873_si_001.pdf]

Supporting information for: Are ammonia sensors ready for outdoor use?

Pablo Espina-Martin[1*], Sarah R. Leeson[1], Robert Nicoll[1], Clare Pearson[1], Cristina Martin Hernandez[1], Nathalie Redon[2], Neil J. Mullinger[1], Karen Yeung[1], Marsailidh M. Twigg[1], Ajinkya G. Deshpande[1], Matthew R. Jones[1], Hilary Costello[3], Graham Spelman[3], Christine F. Braban[1]

1: UK Centre for Ecology and Hydrology, Bush Estate, Penicuik EH26 0QB, UK
2 :IMT Nord Europe, Institut Mines-Télécom, Univ. Lille, Centre for Energy and Environment, F-59000 Lille, France
3: Limosaero Limited, 11c Alma Road, Snettisham, King's Lynn, England, PE31 7NY

Corresponding author*: Pablo Espina Martin, pabesp@ceh.ac.uk,

This file includes supporting materials to the manuscript entitled "*Are ammonia sensors ready for outdoor use*?"**.** The following items can be found in this document:

- 12 pages containing 7 subsections.
- 11 figures: S1 to S11
- 6 tables: S1 to S6

S1. NH_3 sensors selected discarded from advancing to the experimental intercomparison in Whim Bog

Table S1. Details of the discarded NH_3 sensors during the market research phase.

Sensor	Supplier	NH_3 range (ppm)	LOD (ppm)	Sensitivity (nA ppm^{-1})	Accuracy (%)	Resolution (ppm)	Response time (s)	Linearity (%)
EVALAN	EVALAN	0-100	n. p	n. p	±5	0.01	n. p	n. p
NH_3 3E 100	Sensoric	0-100	n. p	90 ± 40	n. p	< 2	at 20 C<br>t_{50} <20s<br>t_{90} < 120s	<5
NH_3 3E 100 SE	Sensoric	0 - 100	n. p	130 ± 30	n. p	< 2	t_{50} <20s<br>t_{90} < 60s	<10
NH4-NH_3	Nemoto	0-100	1	40 ± 12	10	n. p	t_{90} <90 s	n. p
FECS44-100	FIGARO	0-100	n. p	100 ± 30	10	1	60s	n. p
NH_3-B1	ALPHASENSE	0-100	n. p	20-60	n. p	n. p	t_{90}<150	±5
NH_3-AF2	ALPHASENSE	0-100	n. p	15 – 40	n. p	n. p	t_{90} <150	±5
NT-NH_3-PL100	N.E. T	0-100	n. p	10	n. p	1	t_{90} <60	n. p
50 ppm NH_3	Prana Air	0-50	0.0003	n. p	n. p	0.01	< 30	n. p
MQ-135	Waveshare	10-300	n. p	n. p	n. p	n. p	n. p	n.p

n.p: not presented in the datasheets or exchanges with the supplier

.

S2. Set-up of the sensor calibration box and Whim Bog experiment

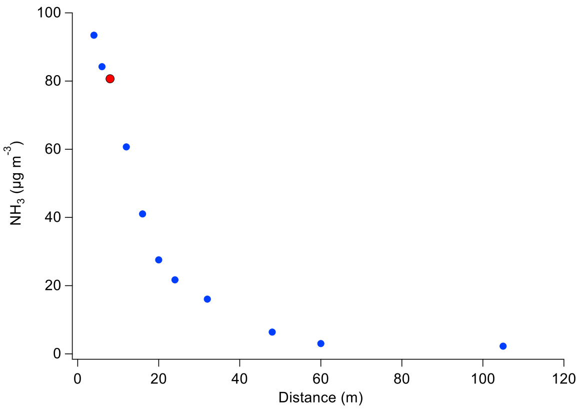

Figure S1. 2002-2022 average NH_3 concentration gradient measured in Whim Bog at 0.5 m heigh above the ground using ALPHA samplers. The red marker corresponds to the concentration measured at the distance where the Picarro and sensor boxes were installed. Source: Deshpande et al. (2024).

Deshpande, A.G., Jones, M.R., Leeson, S.R., Harvey, D., van Dijk, N., Grenier, M., Duarte, F., Stephens, A., Iwanicka, A., Sheppard, L.J., Sutton, M.A., Cape, J.N., Leith, I.D., Levy, P., 2024. Ammonia concentration and deposition data from a peatland nitrogen pollution experiment, Whim Bog, Scotland, UK, 2002-2022 [WWW Document]. EIDC. URL https://catalogue.ceh.ac.uk/id/0cfcde23-753d-4bf0-81c6-13aea33b2b0f (accessed 3.4.25).

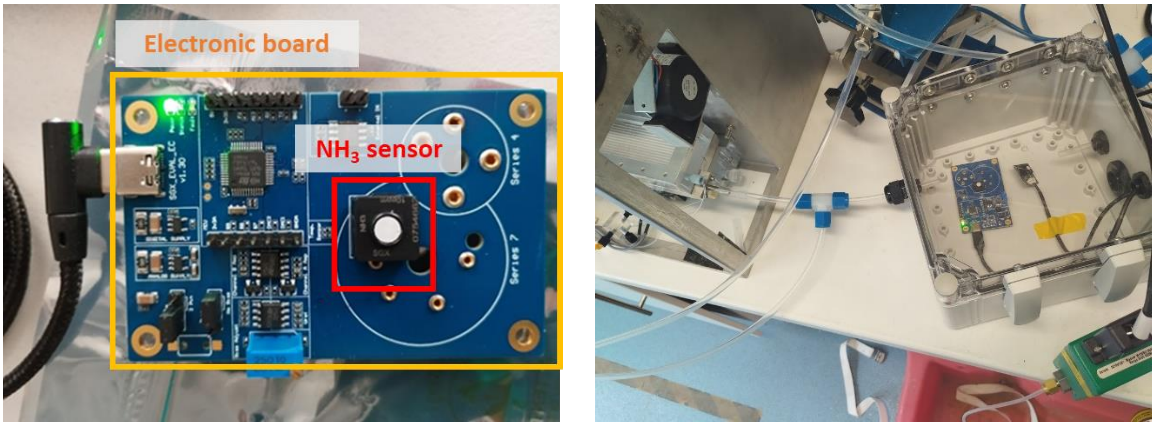

Figure S2.(left) PS1 sensor elements (right) Sensor test chamber used to calibrate the PS1 sensor.

S3. Picarro calibration set up and curves pre and post campaign

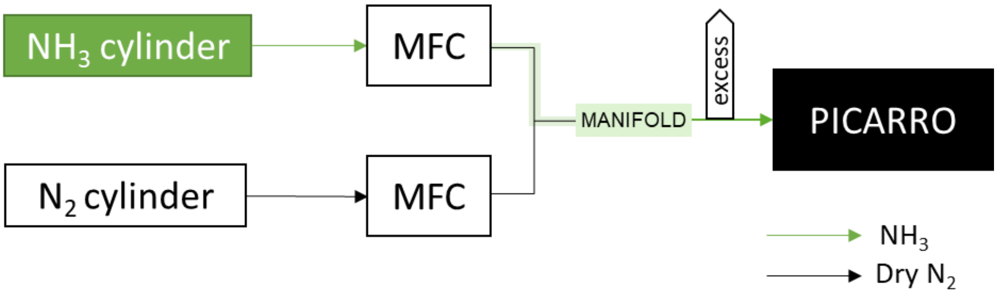

Figure S3. Schematic view of the set-up used for the reference instrument linearity check.

Table S2. Concentration levels used in the Picarro linear check pre and post field deployment.

Theoretical NH_3 concentration (ppb)	Measured NH_3 concentration (ppb)	
	Pre-campaign	Post-campaign
2857.1	2546	2482.9
2142.9	1924	1895.8
1428.6	1282.9	1294
714.3	640.2	649.5
142.9	84.3	134.5
71.4	n.a	46.4
7.1	3.1	n.a

The Picarro uncertainty has been estimated[19] to be 5% if NH_3>10 ppb, and 7% if $NH_3 \leq$10ppb when the analyzer is sampling through a manifold and using an external pump at 1.5 l min^{-1}. The values for each concentration level were calculated as the average of at least a 30-minute period when the Picarro NH_3 concentration had stabilized, following 45 minutes of the standard gas being supplied to the analyzer. The slopes of the theoretical to measured concentrations were 0.9 and 0.87 before and after the deployment, respectively. This underestimation between 10-13% of the theoretical NH_3 concentrations most likely comes from losses in the long inlet line (≈ 3m) used to connect the NH_3 gas bottle located in a non-insulated gas cage and the steel MFCs used to dilute the N_2 and NH_3 mixtures as adsorption resistant coated MFCs were not available.

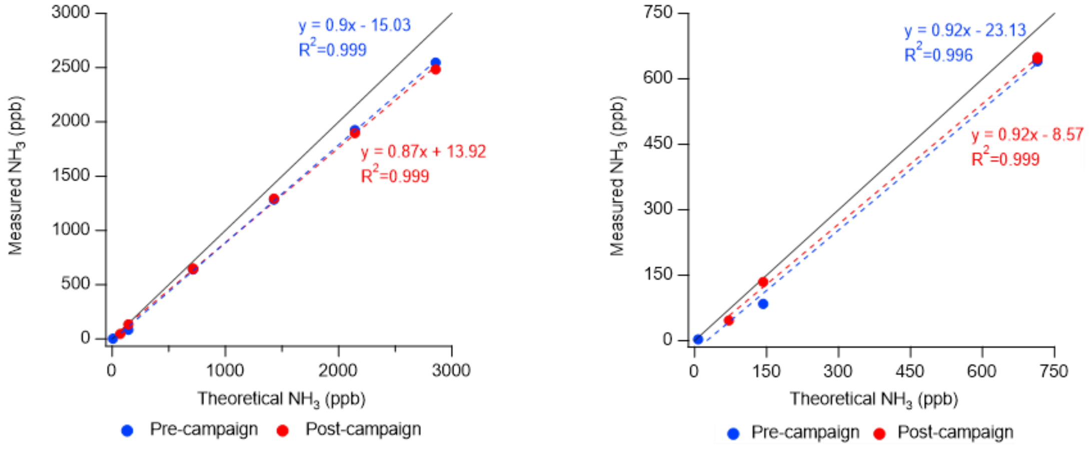

Figure S4. Linear check of the Picarro NH_3 analyzer pre campaign and post campaign for all linear range (left) and for the lowest three concentration levels (right). The solid black line represents the 1:1 line.

S4. MELBA data acquisition

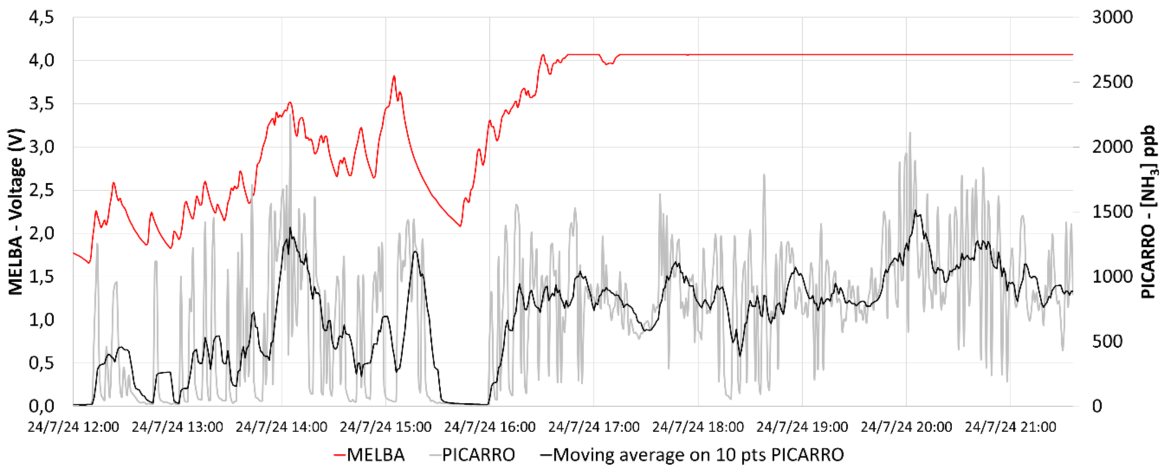

Figure S5. MELBA corrected time series (in V) at the start of the campaign compared against the Picarro NH_3 analyzer (ppb NH_3).

MELBA sensors are based on conducting polymer chemo-resistive surfaces and were developed by IMT Nord Europe. They are equipped with an internal fan to improve diffusion of the air samples contacting the sensitive surface at a 0.2 l min^{-1} rate; the speed of the fan can be modified to change the resulting airflow. The MELBA data corrects the influence of RH using a Kalman filter and then, the absolute concentrations through means of linear regression against a reference analyzer.

They were deployed on the field for the first time during this campaign inside the sensor box containing the PS1 and TB600B (Figure 1d). The MELBA sensor dynamic raw response is progressive and damped, with a trend similar to the Picarro's averaged for every 10 min. After a few hours, a saturation effect can be observed, due to the fact that this sensor is calibrated in laboratories conditions for concentrations below 200 ppb and RH<80% (Table S4).

The sensor is calibrated at 11%, 30% and 40% RH. Although this calibration is valid up to 85% RH, during the evenings and nighttime of the campaign the RH rose up to 95 %. The combination of very high concentrations and high RH values made the sensor drift after a couple of hours at the very beginning of the comparison period. While some parameters can be set (offset and gain), further adjustments are therefore necessary to adapt to more demanding real-world conditions.

S5. Scentroid intercomparison between LC and HC sensors

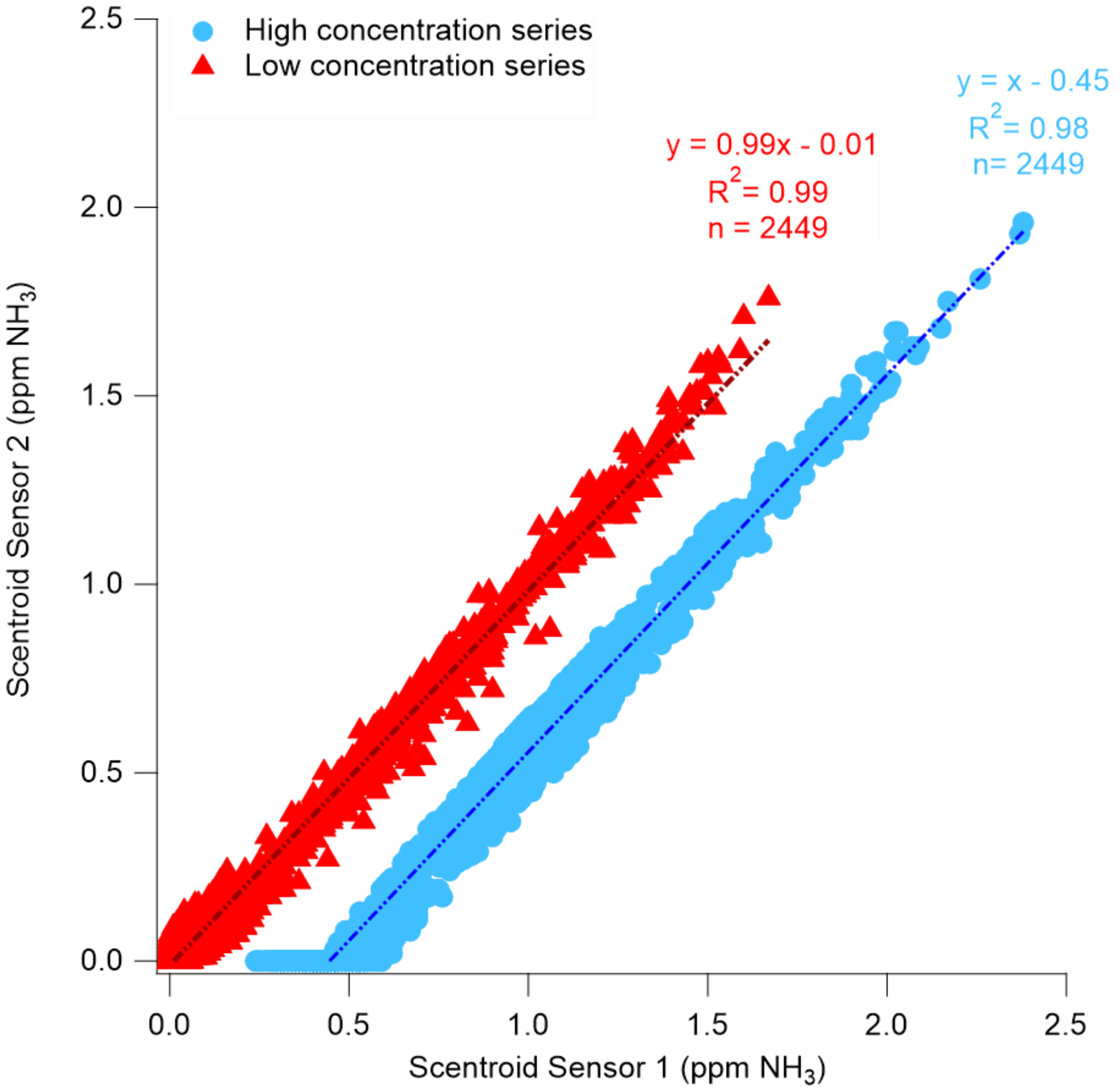

Figure S6. Comparison between LC and HC duplicated sensors.

Table S3. Linear regression parameters between the duplicated HC and LC sensors.

Sensor	Slope ± bias	Offset± bias	R^2	N data	Standard error
HC1/HC2	1 ± 0.003	-0.45 ± 0.01	0.98	2449	0.06
LC1/LC2	0.99 ± 0.002	-0.01 ± 0.001	0.98	2449	0.03

S6. Influence of T and RH on sensors performance during the field campaign

Table S4. Manufacturer specifications compared to the working conditions observed at Whim bog during the intercomparison. Values in bold correspond to the optimal performance conditions specified by the manufacturer. Values in cursive are the conditions where sensors performance have been assessed.

Sensor	Temperature (°C)		RH (R%)		NH_3 range (ppm)	
	Supplier	Whim	Supplier	Whim	Supplier	Whim
Picarro	-10 – 45	3.5 – 22.3	<99	38 -99.7	0 - 1000	0 -3.5
TB600B	-20 -55<br>**25**	3.5 – 22.3	15-95<br>**50**	38 -99.7	0-10	0 -3.5
PS1	-40 – 55<br>*20*	3.5 – 22.3	15 – 95<br>*50*	38 -99.7	0-10	0 -3.5
ECtox	-20 -55<br>**25**	3.5 – 22.3	15-95<br>**50**	38 -99.7	0 -50	0 -3.5
AM HC	5 – 40<br>*23.2*	3.5 – 22.3	10 – 90<br>*45*	38 -99.7	3 – 100	0 -3.5
AM LC	5 – 40<br>*23.2*	3.5 – 22.3	10 – 90<br>*45*	38 -99.7	0.005 - 10	0 -3.5
MELBA	-5-50	3.5 – 22.3	20-80<br>*11, 30, 40*	38 -99.7	0 – 0.5<br>*0.2*	0 -3.5

Influence of T (°C) against NH_3 concentrations (ppm) measured by the sensors

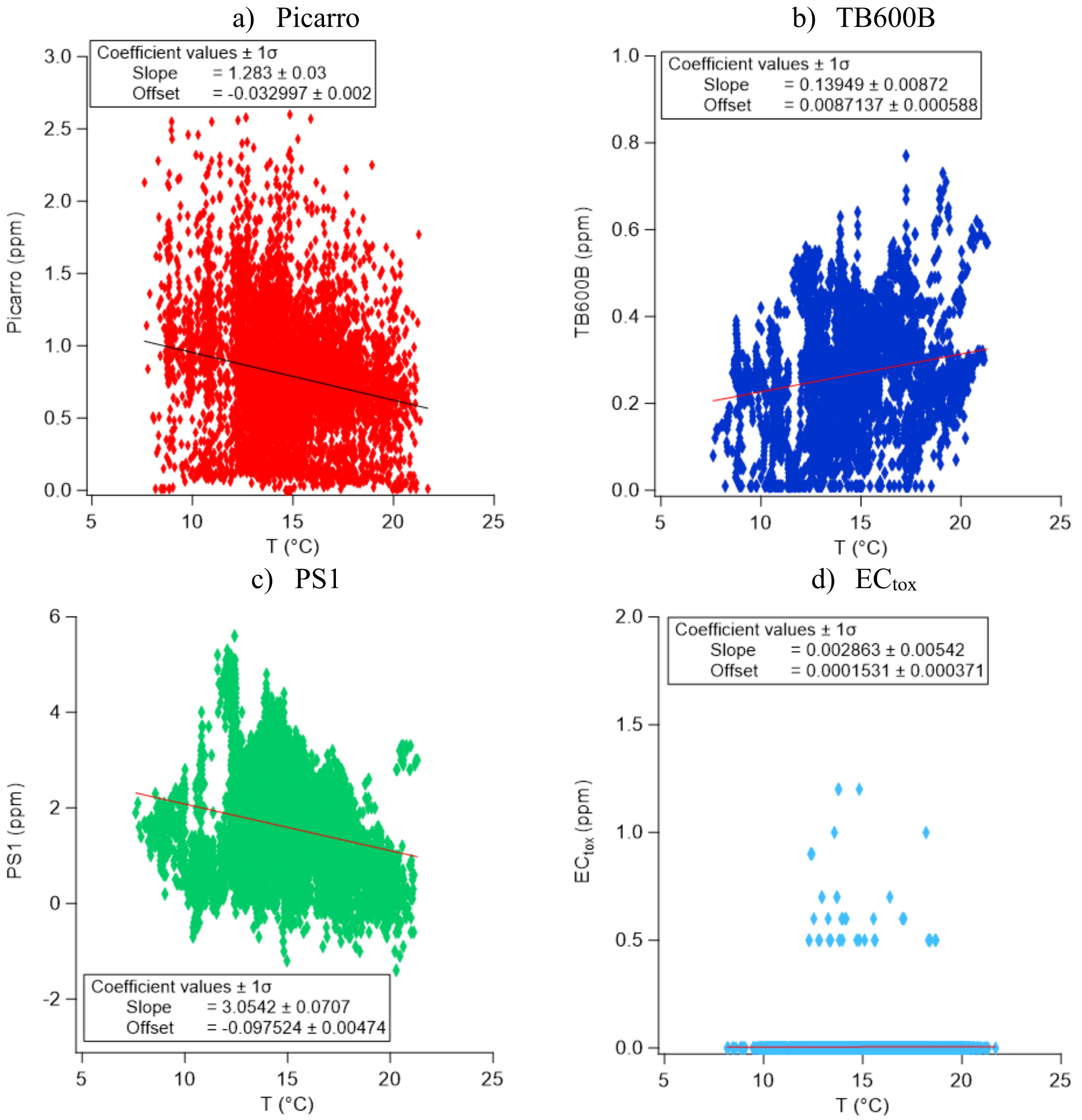

Figure S7. Linear regressions of T (°C) against the NH_3 (ppm) measured by the Picarro (a), TB600B (b), PS1 (c), EC_{tox} (d).

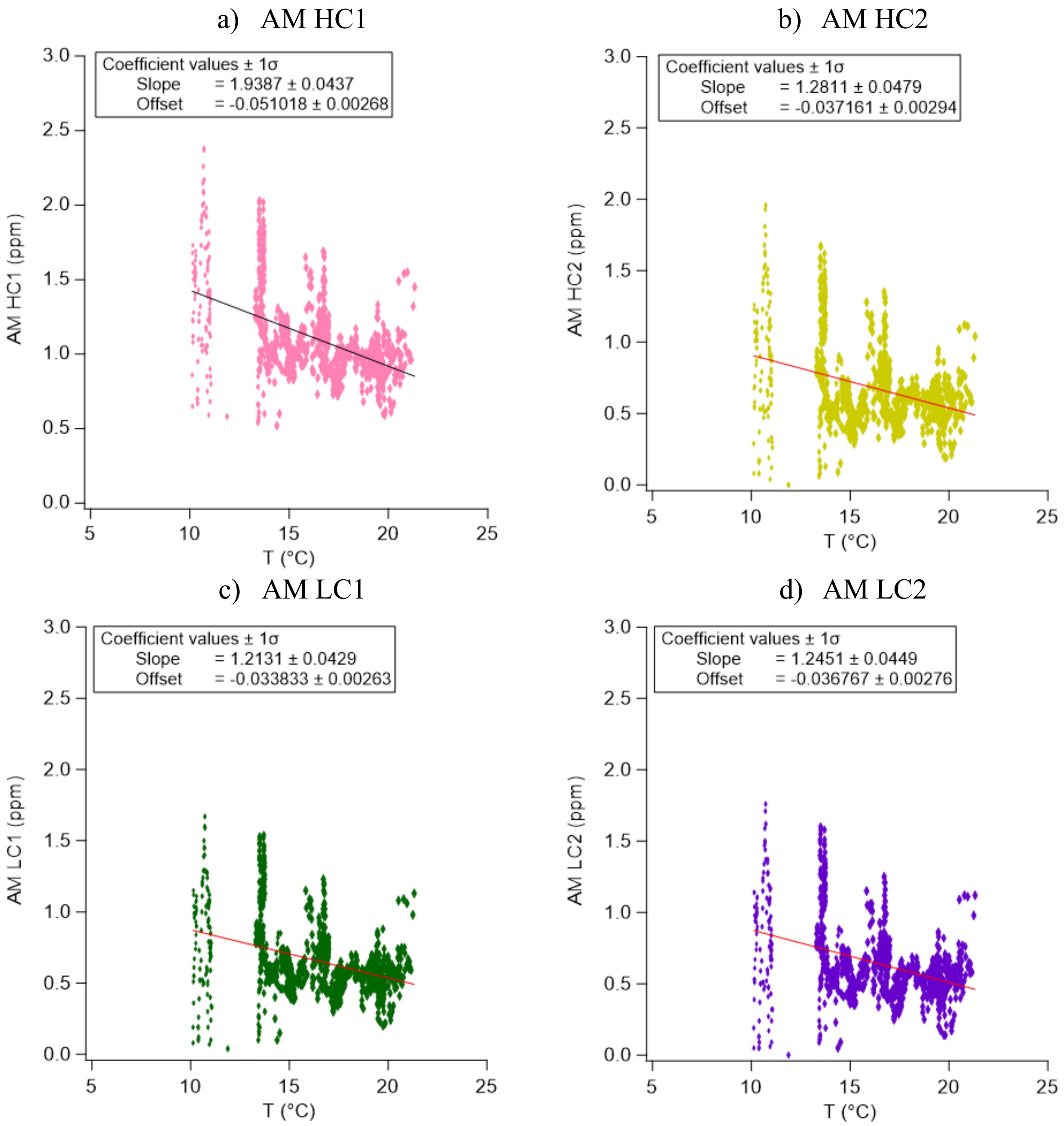

Figure S8. Linear regressions of T (°C) against the NH_3 (ppm) measured by the AM sensors: HC1 (a), HC2 (b), LC1 (c), LC2 (d).

Influence of RH against NH_3 concentrations (ppm) measured by the sensors

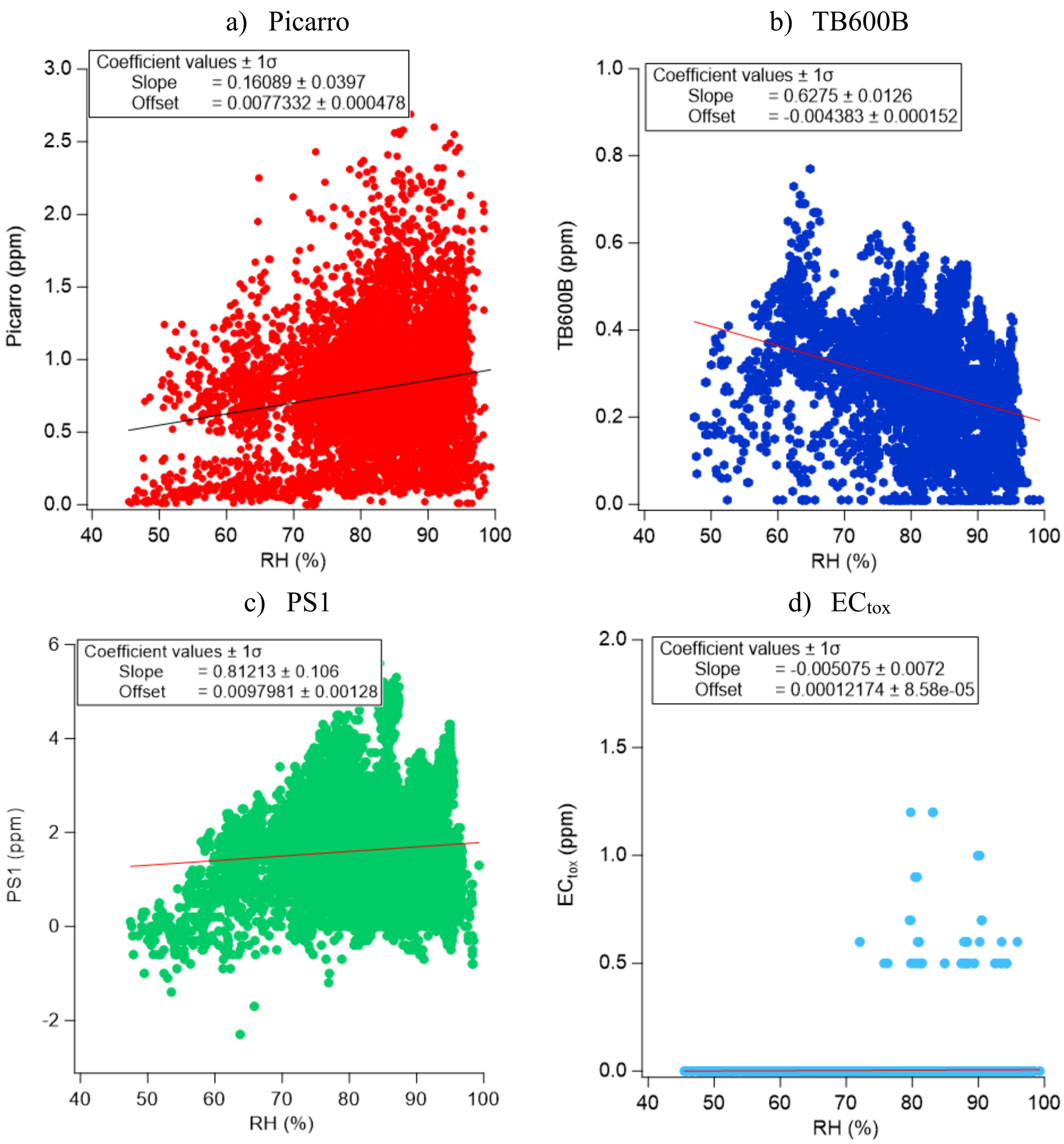

Figure S9. Linear regressions of RH (%) against the NH_3 concentrations (ppm) measured by the Picarro (a), TB600B (b), PS1 (c), ECtox (d).

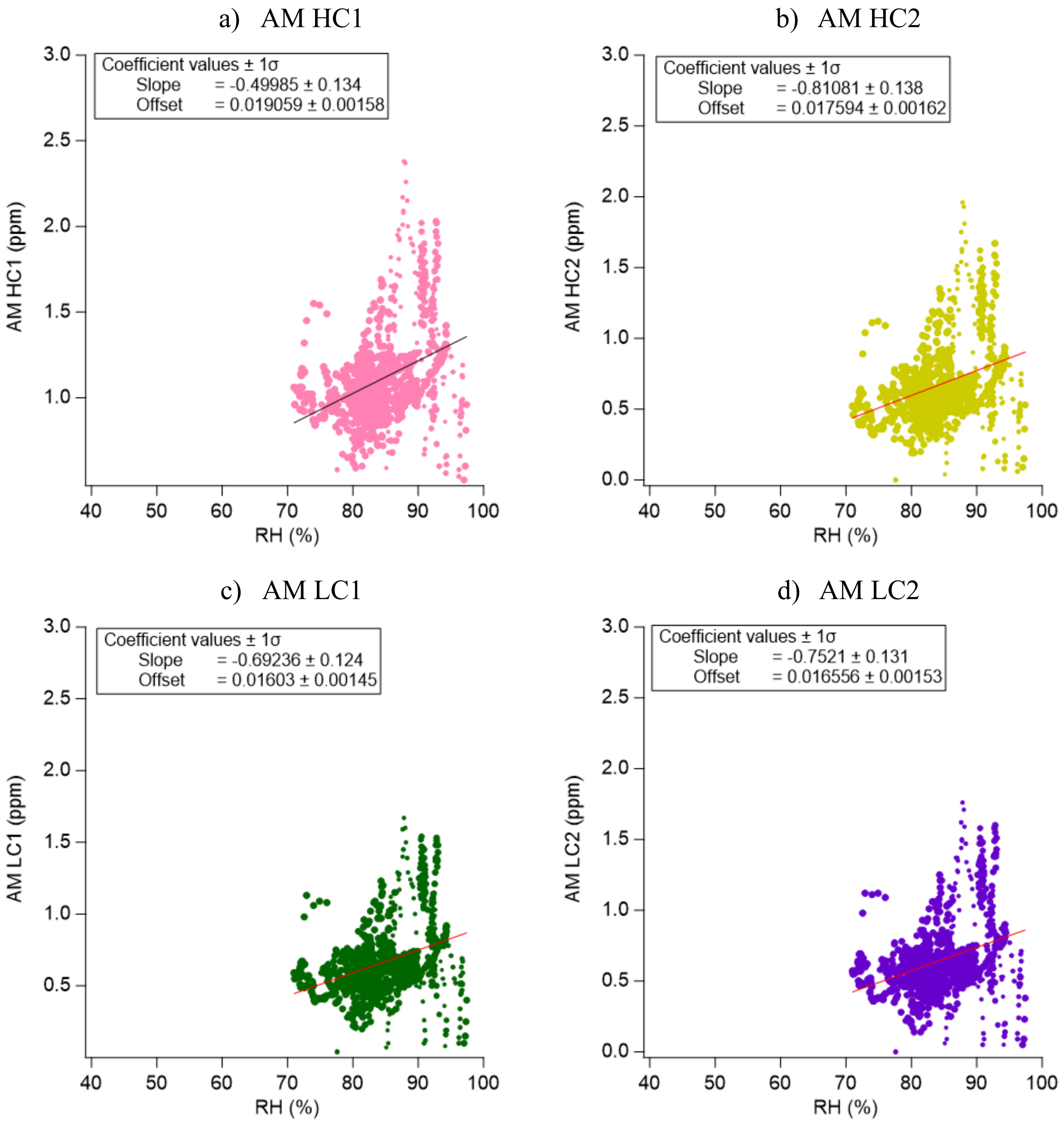

Figure S10. Linear regressions of RH (%) against the NH_3 (ppm) measured by the AM sensors: HC1 (a), HC2 (b), LC1 (c), LC2 (d).

S7. Changes in the sensor housing and NH_3 concentration gradient between outdoors and in-box

Table S5. NH_3 concentrations of the Picarro, TB00B, and I & O-ALPHAs during the exposure periods.

	Period 1 (29/07- 05/08)	Period 2 (05/08- 12/08)	Period 3 (12/08-19/08)	Period 4 (19/08-24/08)
PICARRO (µg m^{-3})	115.25	151.31	144.13	167.14
TB600B (µg m^{-3})	45.84	77.42	61.76	37.54
O-ALPHAs (µg m^{-3})	48.77	91.42	91.01	107.11
I-ALPHAs (µg m^{-3})	n.a.	59.46	68.31	102.77

Table S6. NH_3 ratios between Picarro,TB00B, and I & O-ALPHAs during the exposure periods.

	Period 1 (29/07- 05/08)	Period 2 (05/08- 12/08)	Period 3 (12/08-19/08)	Period 4 (19/08-24/08)
Picarro/TB600B	2.51	1.95	2.33	4.45
O-ALPHA/ Picarro	0.42	0.60	0.63	0.64
I-ALPHA/ Picarro	n.a.	0.39	0.47	0.61
O-ALPHA/ TB600B	1.06	1.18	1.47	2.85
I-ALPHA/ O-ALPHA	n.a.	0.65	0.75	0.96

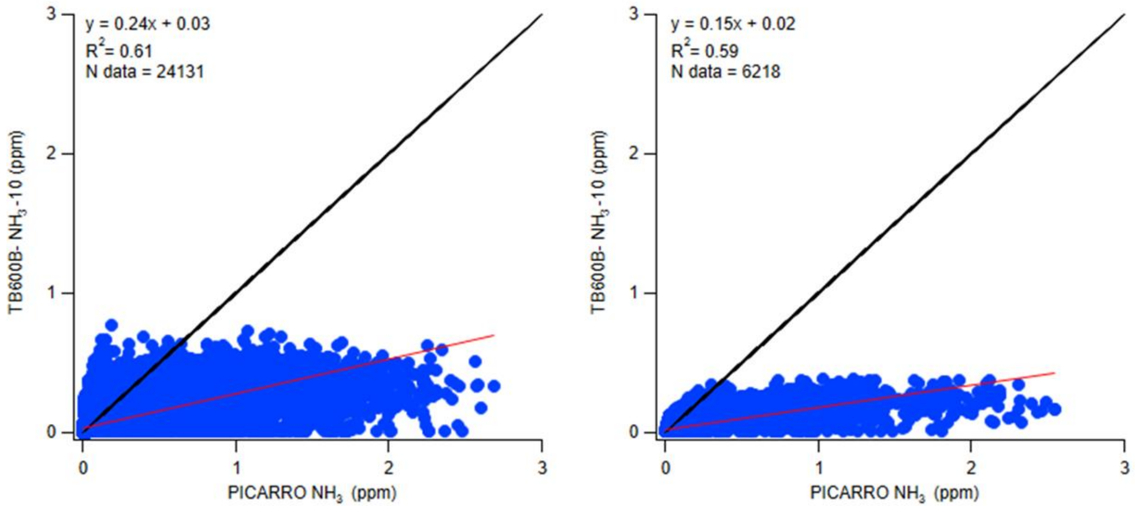

Figure S11. Linear regression between the TB600B and Picarro analyzer NH_3 concentrations when the SH external pump was at 5.5 l min^{-1} (left) and 10 l min^{-1} (right) airflow. The black line corresponds to the 1:1 line, the red line corresponds to the linear fit for the sensor and Picarro data.
